# Clinical Assessment of Thromboembolic Risk in Patients Undergoing Elective Electrical Cardioversion with or Without Transesophageal Echocardiography: A Real-World Observational Study

**DOI:** 10.3390/medicina62050970

**Published:** 2026-05-15

**Authors:** Ana Petretić, Fabio Kadum, Paulina Kušan, Gordana Žauhar, Lara Batičić, Robert Bernat

**Affiliations:** 1 Clinic for Cardiovascular Diseases, University Hospital Center Rijeka, 51000 Rijeka, Croatia; aatonic326@gmail.com (A.P.); kadum.fabio@gmail.com (F.K.); 2 Faculty of Medicine, University of Rijeka, 51000 Rijeka, Croatia; paulina.kusan@gmail.com; 3 Department of Medical Physics and Biophysics, Faculty of Medicine, University of Rijeka, 51000 Rijeka, Croatia; gordana.zauhar@uniri.hr; 4 Department of Medical Chemistry, Biochemistry and Clinical Chemistry, Faculty of Medicine, University of Rijeka, 51000 Rijeka, Croatia; 5 Clinic for Internal Medicine 2, Cardiology, Pulmonology, Angiology and Internal Intensive Care Medicine, Westpfalz-Klinikum Kaiserslautern, 67655 Kaiserslautern, Germany; robert.bernat@icloud.com; 6 Faculty of Medicine, University of Osijek, 31000 Osijek, Croatia

**Keywords:** atrial fibrillation, electrical cardioversion, transesophageal echocardiography, thromboembolism

## Abstract

*Background and Objectives:* Elective electrical cardioversion (eECV) in patients with atrial fibrillation (AF) or atrial flutter is associated with a low risk of thromboembolic events (TEs) when adequate anticoagulation is provided. However, the role of routine transesophageal echocardiography (TEE) prior to eECV remains uncertain. This study aimed to assess thromboembolic outcomes in patients undergoing eECV with or without TEE guidance in a real-world clinical setting. *Methods:* A single-center, combined retrospective–prospective observational study including 296 adequately anticoagulated patients with AF or atrial flutter scheduled for eECV was conducted. The retrospective cohort (n = 220) underwent eECV without routine TEE, while the prospective cohort (n = 85) underwent TEE prior to eECV. The primary outcome was the occurrence of thromboembolic events (ischemic stroke or systemic embolism) within 30 days after eECV. Arrhythmia recurrence at 30 days was assessed as a secondary, exploratory outcome. *Results:* Among patients undergoing eECV, thromboembolic events were rare. In the retrospective cohort, 3/220 patients (1.36%) experienced a TE, whereas no events were observed in the prospective cohort (0/76). Due to the low number of events, the study was underpowered to detect meaningful differences between strategies (RR 2.44; 95% CI 0.13–46.7; *p* = 0.55). TEE identified left atrial appendage thrombus in 9/85 screened patients (10.6%), who were subsequently excluded from cardioversion. Arrhythmia recurrence at one month was more frequent in the prospective cohort (19.7% vs. 7.3%), likely reflecting differences in baseline clinical characteristics. *Conclusions:* Thromboembolic events after eECV in adequately anticoagulated patients were infrequent in this real-world cohort. While the study design limits direct comparison between strategies, the results indicate that a conventional anticoagulation-based approach without routine TEE is associated with a low risk of thromboembolic events in most patients. At the same time, the detection of left atrial appendage thrombus in a subset of patients underscores the importance of individualized risk assessment and supports the selective use of TEE in higher-risk clinical settings.

## 1. Introduction

Atrial fibrillation (AF) is one of the most common persistent cardiac arrhythmias and cardiovascular disorders, representing a significant public health burden [1,2]. The costs of AF management are substantial and long-term [3]. AF is associated with an increased risk of mortality, hospitalization, stroke, heart failure (HF), and cognitive decline [1,4].

To improve patient care, an integrated management model known as AF-CARE has been proposed (C—control of risk factors and comorbidities; A—avoidance of stroke and thromboembolism; R—reduction of symptoms through rhythm and rate control; E—evaluation and dynamic reassessment) [1]. Particular emphasis is placed on stroke prevention and symptom control, which are key determinants of prognosis and quality of life. Stroke and HF remain leading causes of morbidity and mortality in patients with AF [5].

For stroke prevention, individual thromboembolic risk is assessed using the CHA_2_DS_2_-VA score [1]. Oral anticoagulation (OAC) is recommended in patients with a score ≥ 2 and should be considered in those with a score of 1. Direct oral anticoagulants (DOACs)—apixaban, dabigatran, edoxaban, and rivaroxaban—are preferred over vitamin K antagonists, except in patients with mechanical heart valves or moderate-to-severe mitral stenosis. Their efficacy and safety have been demonstrated in multiple randomized controlled trials [6]. The initiation of OAC requires an individualized approach, taking into account patient characteristics, comorbidities, and preferences.

After adequate anticoagulation has been established and in clinically stable patients, strategies for rhythm and/or rate control are considered. The choice of strategy is guided primarily by symptom burden, commonly assessed using the modified European Heart Rhythm Association (EHRA) classification [7]. Rhythm control may be achieved using antiarrhythmic drugs, electrical cardioversion (ECV), or catheter ablation [8].

Synchronized ECV is typically an elective procedure performed in stable patients under short-acting intravenous sedation (e.g., propofol, midazolam, or etomidate), during which an electrical shock is delivered to restore sinus rhythm [8]. However, ECV should only be performed in patients with a low risk of thromboembolism (TE), as the presence of a left atrial appendage (LAA) thrombus represents a contraindication [1,9].

In inadequately anticoagulated patients, TEs may occur within 30 days after ECV in approximately 5–7% of cases [9]. With appropriate anticoagulation, this risk is reduced to less than 1% [10]. Nevertheless, TE risk is not completely eliminated, even in patients with low CHA_2_DS_2_-VA scores or after exclusion of LAA thrombus by transesophageal echocardiography (TEE). Post-cardioversion atrial stunning may contribute to thrombus formation [9].

Therefore, proper patient preparation and adequate anticoagulation are essential. In hemodynamically stable patients with AF of unknown duration, elective ECV (eECV) is recommended only after at least 3 weeks of effective anticoagulation. Alternatively, TEE may be performed prior to eECV to exclude LAA thrombus [1,8,9]. Both strategies are supported by current guidelines and are associated with a low risk of TEs, generally below 1% [9,10].

Despite this, many centers routinely perform TEE prior to eECV, even in adequately anticoagulated patients [11]. This practice is supported by evidence showing that LAA thrombi may still be detected by TEE or cardiac computed tomography in such patients [12]. Several clinical conditions, including heart failure, diabetes, malignancy, and systemic inflammation, are associated with an increased risk of thrombus formation [4]. However, the clinical value of routine TEE in adequately anticoagulated patients remains uncertain, particularly in real-world settings.

The aim of this study was to assess thromboembolic outcomes in adequately anticoagulated patients with atrial fibrillation or atrial flutter undergoing elective electrical cardioversion, in the context of two real-world clinical management strategies: a conventional approach based on guideline-recommended anticoagulation without routine transesophageal echocardiography, and a transesophageal echocardiography-guided approach. The study also aimed to provide descriptive insight into the prevalence of left atrial appendage thrombus and to highlight the importance of individualized risk assessment in routine clinical practice.

## 2. Materials and Methods

This study was designed as a single-center, combined retrospective–prospective observational study conducted at the Clinic for Cardiovascular Diseases, Clinical Hospital Center Rijeka. A total of 296 patients with atrial fibrillation or atrial flutter who were scheduled for elective electrical cardioversion between January 2023 and October 2025 were included in the study. The primary objective of the study was to evaluate thromboembolic outcomes in adequately anticoagulated patients undergoing elective electrical cardioversion, within the context of two different real-world clinical management strategies. The study was not designed to compare directly equivalent patient populations, but rather to reflect routine clinical decision-making and practice patterns. No formal sample size calculation was performed due to the exploratory nature of the study.

Two cohorts were analyzed: (1) a retrospective cohort of patients who underwent elective electrical cardioversion without routine use of transesophageal echocardiography, representing a conventional approach based on guideline-recommended anticoagulation; and (2) a prospective cohort of patients managed with a transesophageal echocardiography-guided strategy prior to cardioversion, in which additional imaging was performed despite adequate anticoagulation, based on clinical judgment. These two cohorts represent distinct management strategies rather than matched groups, and are therefore inherently non-comparable. In the transesophageal echocardiography-guided cohort, patients with detected left atrial appendage thrombus were excluded from cardioversion in accordance with current guidelines, resulting in a lower-risk population proceeding to the procedure. This reflects real-world clinical practice but introduces an important source of selection bias, which is acknowledged in the analysis and interpretation.

Inclusion criteria were patients aged ≥18 years with a diagnosis of atrial fibrillation or atrial flutter who were scheduled for elective electrical cardioversion as part of a rhythm control strategy and who were adequately anticoagulated.

Adequate anticoagulation was defined as continuous and regular oral anticoagulant therapy for at least three weeks prior to cardioversion, including therapeutic doses of dabigatran, rivaroxaban, apixaban, edoxaban, or warfarin, in accordance with current clinical guidelines (Table 1). For patients treated with warfarin, anticoagulation was considered adequate if the international normalized ratio was maintained between 2.0 and 3.0 in at least two consecutive measurements prior to the procedure.

All patients underwent standard clinical evaluation prior to cardioversion, including assessment of medical history, comorbidities, and anticoagulation status. In the retrospective cohort, data were obtained from the hospital information system, with additional follow-up information collected when necessary. In the prospective cohort, patients were evaluated according to a predefined clinical protocol, including transesophageal echocardiography performed prior to cardioversion.

### 2.1. Exclusion Criteria and Study Procedures

Exclusion criteria included hemodynamically unstable patients requiring urgent cardioversion, known contraindications to TEE (ascending aortic dilatation, esophageal strictures, varices, or prior esophageal surgery), and pregnancy. The retrospective cohort comprised 220 patients who underwent eECV without TEE. Data were collected from the Integrated Hospital Information System, and missing data were obtained via telephone follow-up. The prospective cohort included 85 patients who underwent TEE prior to eECV. Of 85 screened patients, 9 were excluded due to LAA thrombus, resulting in 76 patients included in the final analysis. The study was approved by the Ethical Committees and all patients provided written informed consent. Before the procedure, the responsible cardiologist explained both strategies, including their benefits and potential risks. All patients were screened for inclusion and exclusion criteria prior to enrollment.

TEE was typically performed on the same day as the planned eECV following appropriate patient preparation (fasting state, intravenous cannula placement—usually in the cubital vein—and administration of 5–10 mg intravenous diazepam according to body weight, along with topical lidocaine spray). TEE was performed using a transesophageal probe on a cardiovascular ultrasound system (GE Healthcare Vivid E95 3D/4D) by cardiologists specialized in cardiovascular imaging.

Patients in whom a left atrial appendage (LAA) thrombus was detected were not cardioverted and were excluded from outcome analysis but were reported descriptively.

The primary outcome was the occurrence of thromboembolic events (TEs), defined as ischemic stroke or systemic embolism within one month after eECV, comparing a conventional anticoagulation-based strategy with a TEE-guided strategy. The secondary outcome was arrhythmia recurrence at one month. In the retrospective cohort, outcomes were assessed from medical records. In the prospective cohort, 30-day outcomes were assessed during scheduled follow-up visits.

Additional variables included sex (male/female), age (years), body weight (kg), height (cm), and body mass index (BMI, kg/m^2^). Arrhythmia type was classified as paroxysmal AF, persistent AF, long-standing persistent AF, paroxysmal atrial flutter, persistent atrial flutter, or long-standing persistent atrial flutter. Symptoms were categorized as asymptomatic, palpitations, dyspnea, or chest pain. Symptom severity was assessed using the European Heart Rhythm Association (EHRA) classification (I–IV).

Left atrial (LA) size (parasternal short-axis view) was categorized as <40 mm, 40–49 mm, 50–59 mm, or ≥60 mm. Comorbidities included arterial hypertension, diabetes mellitus, heart failure (HFpEF, HFmrEF, HFrEF), chronic kidney disease (G1–G5), vascular disease (coronary artery disease or peripheral arterial disease), obstructive sleep apnea, prior stroke or transient ischemic attack, smoking status, and COVID-19 vaccination status.

Additional procedural variables included type of anticoagulation, CHA_2_DS_2_-VA score, type of antiarrhythmic premedication (none, amiodarone, propafenone, flecainide), sedation dose (propofol, mg), number of eECV attempts, maximum delivered energy (J), and restoration of sinus rhythm (yes/no).

### 2.2. Statistical Analysis

Statistical analysis was performed to describe and explore differences between the two management strategies, recognizing the observational nature of the study. Continuous variables are presented as mean ± standard deviation or median with interquartile range, depending on data distribution. Comparisons between groups (patients undergoing cardioversion with or without transesophageal echocardiography) were performed using Student’s *t*-test or the Mann–Whitney U test, as appropriate. Categorical variables are presented as counts and percentages. Differences between groups were analyzed using the chi-square test or Fisher’s exact test, as appropriate. Fisher’s exact test was used in cases with small sample sizes or when expected cell counts were low, including analyses involving thromboembolic events. The incidence of thromboembolic events and arrhythmia recurrence is reported as proportions with corresponding 95% confidence intervals. Relative risks with 95% confidence intervals are provided for descriptive purposes only. Given the low number of events and the non-randomized study design, the analysis is primarily descriptive and exploratory. No multivariable analysis was performed due to the limited number of outcome events and the risk of overfitting. A two-sided *p*-value < 0.05 was considered statistically significant.

## 3. Results

In the prospective cohort (n = 85), left atrial appendage (LAA) thrombus was identified in 9 patients (10.6%; 95% CI, 0.05–0.20), who were subsequently excluded from cardioversion. In the retrospective cohort (n = 220; mean [SD] age, 68.9 [7.6] years; 33.6% women), three patients (1.36%; 95% CI, 0.002–0.03) experienced a thromboembolic event (TE) despite adequate anticoagulation. One patient experienced an ischemic stroke and one acute mesenteric ischemia (both classified as thromboembolic events), while one patient experienced a myocardial infarction (reported separately).

In the prospective cohort (n = 76; mean [SD] age, 66.9 [9.6] years; 40.8% women), no patients experienced TE (0%; 95% CI, 0.0–0.039). The relative risk was 2.44 (95% CI, 0.13–46.7; *p* = 0.55). There were no statistically significant differences between the cohorts (*p* = 0.31). No statistically significant differences in thromboembolic event (TE) incidence were observed between the retrospective and prospective cohorts (*p* = 0.550). In total, three TEs (1.01%) occurred, all within the retrospective cohort (3/220; 1.36%), while no events were observed in the prospective cohort (0/76). These findings suggest a low overall incidence of TE following eECV in adequately anticoagulated patients, consistent with a low overall event rate across both management strategies (Table 2).

In contrast, a statistically significant difference was observed in arrhythmia recurrence at one month between the cohorts (*p* = 0.002). Arrhythmia recurrence occurred in 31 patients (10.5%) overall, with a higher rate in the prospective cohort (15/76; 19.7%; 95% CI, 0.11–0.30) compared with the retrospective cohort (16/220; 7.27%; 95% CI, 0.04–0.11). This finding may reflect differences in patient selection or clinical characteristics between the cohorts.

In the retrospective cohort, 13 patients (5.9%) were lost to follow-up, and eECV was unsuccessful in an additional 13 patients (5.9%).

Most baseline clinical characteristics were comparable between cohorts. However, statistically significant differences were observed in symptom distribution, EHRA class, and prevalence of chronic kidney disease (Table 3). Patients in the prospective cohort were more symptomatic, with a higher proportion of dyspnea and combined symptoms, and were more frequently classified as EHRA class 2a or higher. Chronic kidney disease was more prevalent in the retrospective cohort.

Within the prospective cohort, patients excluded from cardioversion due to confirmed LAA thrombus (n = 9) were analyzed separately. The mean age was 69.9 ± 11.7 years, with a predominance of men (66.7%). The mean body mass index was 30.4 kg/m^2^, with higher values observed in women compared with men (31.8 vs. 29.7 kg/m^2^). Most patients had persistent atrial fibrillation (88.9%). Two patients (22.2%) were asymptomatic, while dyspnea was the most common presenting symptom (55.6%). According to the EHRA classification, class IIa predominated (55.6%). Data on left atrial size were available for eight patients, with half demonstrating a diameter ≥ 50 mm. Comorbidities were highly prevalent, including arterial hypertension (77.8%), type 2 diabetes mellitus (33.3%), heart failure (77.8%; most commonly HFrEF, 55.6%), and chronic kidney disease (55.6%).

The mean CHA_2_DS_2_-VA score was 3.7 ± 1.9. Notably, LAA thrombus was detected despite treatment with direct oral anticoagulants, most commonly full-dose apixaban and rivaroxaban (Table 4). Dose of oral anticoagulant was checked before eECV in every patient according to the renal function.

## 4. Discussion

In this study, no statistically significant difference in the incidence of thromboembolic events (TEs) was observed between patients undergoing elective electrical cardioversion (eECV) with or without prior TEE. Overall, the rate of TE was low, which is consistent with previously published data reporting rates of approximately 0.6% to 1% after cardioversion in adequately anticoagulated patients [9,10]. These findings are in agreement with current guideline recommendations, which allow eECV without routine TEE in patients who have received at least three weeks of effective anticoagulation [1].

AF is one of the most common arrhythmias worldwide and is associated with a high risk of stroke, systemic embolism, and increased healthcare burden [2,3]. The risk of TE in AF is influenced by multiple factors, including age, comorbidities, and structural heart disease [4,5]. Therefore, optimizing safe and effective strategies for cardioversion remains an important clinical goal. Importantly, the present study should be interpreted as a comparison of two clinical management strategies, rather than a comparison of two directly equivalent patient groups. In the TEE-guided strategy, patients with detected left atrial appendage (LAA) thrombus were excluded from eECV, resulting in a lower-risk population undergoing the procedure. This design inherently reduces observed risk in the TEE group and precludes direct comparison between the two strategies. Consequently, this introduces selection bias and limits the ability to draw causal conclusions regarding their comparative safety. Similar variability in clinical practice and decision-making has been reported in European surveys [11].

A key finding of this study is that TEE identified LAA thrombus in 10.6% of screened patients despite adequate anticoagulation. This prevalence is consistent with previous studies showing that LAA thrombus may still be present in a subset of anticoagulated patients [12,13]. Although modern anticoagulation, including non-vitamin K antagonist oral anticoagulants, has significantly reduced thromboembolic risk [6], it does not completely eliminate it.

However, the presence of LAA thrombus detected by TEE did not translate into a higher rate of clinical TE in the group managed without TEE. This suggests that adequate anticoagulation remains the most important protective factor against TE. Randomized trials evaluating anticoagulation strategies in cardioversion settings have demonstrated very low event rates when anticoagulation is appropriately managed [14,15,16]. These findings support the concept that imaging may not always provide additional clinical benefit in terms of short-term outcomes.

The clinical significance of detecting LAA thrombus in adequately anticoagulated patients remains uncertain. While TEE allows identification of thrombus and may prevent cardioversion in selected cases, it is unclear whether this improves patient outcomes or mainly leads to more conservative management. Similar observations have been reported in patients undergoing catheter ablation, where imaging often detects thrombi despite uninterrupted anticoagulation [17,18,19]. In addition, some studies suggest that thrombus detection may reflect overall patient risk rather than directly predicting short-term TE in all patients [13].

A statistically significant difference in arrhythmia recurrence was observed at one month, with higher recurrence rates in the TEE-guided cohort. However, this finding should be interpreted with caution. It is unlikely that TEE itself affects arrhythmia recurrence. Instead, this difference is most likely explained by baseline differences between the groups. Patients in the prospective cohort had more severe symptoms and higher European Heart Rhythm Association (EHRA) classes [7], indicating more advanced atrial disease. These factors are known to be associated with lower success rates of rhythm control and higher recurrence rates [8].

The relatively high prevalence of LAA thrombus observed in this study highlights the heterogeneity of thromboembolic risk in AF. Even in adequately anticoagulated patients, residual risk may persist due to comorbidities, vascular disease, and underlying atrial remodeling [4,5]. This supports the need for individualized risk assessment rather than a uniform approach to imaging.

Our findings contribute to the ongoing debate regarding the role of routine TEE before cardioversion. While current guidelines support both strategies [1], real-world practice often favors routine imaging due to concerns about safety [11]. However, our results, together with recent real-world studies, suggest that routine TEE may not provide a clear additional benefit in reducing TE in adequately anticoagulated patients [20,21,22]. Instead, it may increase procedural complexity, healthcare costs, and delays in treatment without clear improvement in clinical outcomes.

From a practical perspective, balancing safety and efficiency is essential. The TEE-guided strategy requires additional procedural steps, trained cardiovascular imaging specialists, and may delay eECV. When LAA thrombus is detected, cardioversion is postponed, often for several weeks. This may lead to prolonged symptoms, reduced quality of life, and potential worsening of heart failure.

At the same time, the assumption that TEE universally improves safety should be interpreted cautiously. In this study, no TEs occurred in the TEE-guided cohort; however, this is likely influenced by the exclusion of higher-risk patients. Conversely, the low event rate observed in the non-TEE cohort is consistent with the effectiveness of anticoagulation-based strategies. Although TEE is considered the reference standard for detection of LAA thrombus, its sensitivity is not absolute and may be limited in detecting very small thrombi or in suboptimal imaging conditions. Advanced imaging modalities such as cardiac CT have demonstrated high sensitivity and may complement TEE in selected cases, although their routine use remains limited in clinical practice. Therefore, the absence of thrombus on TEE does not completely eliminate thromboembolic risk. These findings are consistent with contemporary real-world evidence showing low TE risk in adequately anticoagulated patients undergoing cardioversion [20,21,22,23].

In routine clinical practice, standardized steps are important for ensuring efficient and consistent care. While individualized decision-making remains essential, especially in high-risk patients, a strategy based on guideline-recommended anticoagulation without routine TEE appears appropriate for most patients. Selective use of TEE may be reserved for patients with uncertain anticoagulation adherence, high thromboembolic risk, or structural heart disease. Future studies should focus on identifying patients who may benefit most from TEE-guided strategies. In particular, there is a need to better define predictors of LAA thrombus and clinically relevant TE. Large prospective and randomized studies are required to clarify the role of imaging in this setting.

## 5. Conclusions

In adequately anticoagulated patients undergoing eECV, the incidence of thromboembolic events is low both in strategies with and without TEE. However, given the low number of events and the observational design, these findings should be interpreted with caution. Our findings are consistent with current guideline-based strategies that allow cardioversion without routine TEE after at least three weeks of effective anticoagulation. Although TEE identified left atrial appendage (LAA) thrombus in a notable proportion of patients, this did not translate into a higher rate of clinical thromboembolic events in the cohort managed without TEE. This supports the central role of adequate anticoagulation in ensuring procedural safety. At the same time, the detection of LAA thrombus highlights that a subgroup of patients may still carry additional risk that is not fully captured by standard clinical scoring systems. The higher rate of arrhythmia recurrence observed in the TEE-guided cohort is most likely related to differences in baseline clinical characteristics, particularly greater symptom burden and higher EHRA class, rather than the use of TEE itself. Taken together, our results support a selective, individualized approach to TEE use. Routine TEE prior to cardioversion may not be necessary in all adequately anticoagulated patients and should be reserved for specific clinical situations, such as uncertain anticoagulation status, high-risk profiles, or complex structural heart disease.

### 5.1. Limitations

This study has several limitations. The relatively small sample size, particularly in the prospective cohort, limits statistical power to detect differences in rare events such as thromboembolism. The non-randomized design introduces selection bias, as patients undergoing transesophageal echocardiography represent a different clinical population. The exclusion of patients with detected thrombus further contributes to this imbalance. Anticoagulation adherence was assessed based on patient report and may not be fully reliable. In addition, transesophageal echocardiography is subject to interobserver variability. Finally, the relatively short follow-up period may not capture late thromboembolic or arrhythmic events. Due to the low number of events, multivariable analysis was not feasible, limiting adjustment for potential confounders. The study was not designed to identify independent predictors of thromboembolic risk, which should be addressed in future studies.

### 5.2. Future Perspectives

Future research should focus on improving risk stratification beyond current clinical scores, with particular attention to identifying patients who are at increased risk of LAA thrombus despite adequate anticoagulation. Integration of clinical, imaging, and possibly biomarker-based approaches may help define subgroups that would benefit most from TEE-guided strategies. Large, prospective, and ideally randomized studies are needed to determine whether routine or selective TEE use has a measurable impact on clinical outcomes, including thromboembolic events, arrhythmia recurrence, and patient-centered outcomes such as quality of life.

In addition, standardized management protocols for patients in whom LAA thrombus is detected are currently lacking and should be addressed in future studies. Clear guidance on anticoagulation adjustment, timing of repeat imaging, and optimal timing of cardioversion would improve clinical decision-making.

Finally, real-world factors such as available resources, workload, and how healthcare systems are organized should be considered in future research and guidelines, to ensure that recommended strategies are both effective and practical in everyday clinical practice.

## Figures and Tables

**Table 1:** Anticoagulant dosing regimens: dabigatran 150 mg twice daily (BID), or 110 mg BID in patients aged ≥80 years; rivaroxaban 20 mg once daily (OD), or 15 mg OD in patients with moderate to severe renal impairment (creatinine clearance [CrCl] < 50 mL/min); apixaban 5 mg BID, or 2.5 mg BID in patients with severe renal impairment (CrCl 15–30 mL/min) or meeting ≥2 of the following criteria: age ≥ 80 years, body weight ≤ 60 kg, or serum creatinine ≥ 133 µmol/L; edoxaban 60 mg OD, or 30 mg OD in patients with CrCl 15–30 mL/min. Warfarin therapy was considered adequate if the international normalized ratio (INR) was maintained between 2.0 and 3.0 in at least two consecutive measurements. Abbreviations: BID, twice daily; OD, once daily; CrCl, creatinine clearance; INR, international normalized ratio.

Anticoagulant	Standard Dose	Adjusted Dose	Adjustment Criteria
Dabigatran	150 mg BID	110 mg BID	Age ≥ 80 years
Rivaroxaban	20 mg OD	15 mg OD	CrCl < 50 mL/min
Apixaban	5 mg BID	2.5 mg BID	CrCl 15–30 mL/min or ≥2 of: age ≥ 80 years, body weight ≤ 60 kg, serum creatinine ≥ 133 µmol/L
Edoxaban	60 mg OD	30 mg OD	CrCl 15–30 mL/min
Warfarin	Dose adjusted to maintain INR 2.0–3.0	—	INR 2.0–3.0 in ≥2 consecutive measurements

**Table 2:** Clinical outcomes in retrospective and prospective cohorts (TE, thromboembolic event).

Variable	Total (N = 296)	Retrospective (N = 220)	Prospective (N = 76)	*p*-Value
Thromboembolic events (TEs)				0.550
Yes	3	3	0	
No	293	217	76	
Arrhythmia recurrence (1 month)				0.002
Yes	31	16	15	
No	265	204	61	

**Table 3:** Baseline clinical characteristics of the study population (AF, atrial fibrillation; BMI, body mass index; eECV, elective electrical cardioversion; EF, ejection fraction; EHRA, European Heart Association; HF, heart failure; OSA, obstructive sleep apnea).

Variable	Total (N = 296)	Prospective (N = 76)	Retrospective (N = 220)	*p*-Value
Age/years	68.4 ± 8.2	66.9 ± 9.6	68.9 ± 7.6	0.0.64
Gender				
male	191 (64.5%)	45 (59.2%)	146 (66.4%)	0.260
female	105 (35.5%)	31 (40.8%)	74 (33.6%)	0.260
BMI/kg·m^−2^	29.6 ± 5.9	29.9 ± 6.2	29.6 ± 5.7	0.71
Type of arrhythmia				
Persistent AF	242 (81.8%)	62 (81.6%)	180 (81.8%)	0.95
Long-standing persistent AF	9 (3.0%)	2 (2.6%)	7 (3.2%)	0.95
Persistent atrial flutter	44 (14.9%)	12 (15.8%)	32 (14.5%)	0.95
Symptoms				
None	127 (42.9%)	16 (21.1%)	111 (50.5%)	**<0.001**
Palpitations	45 (15.2%)	15 (19.7%)	30 (13.6%)	**<0.001**
Dyspnea	99 (33.4%)	34 (44.7%)	65 (29.5%)	**<0.001**
Palpitations + dyspnea	24 (8.1%)	11 (14.5%)	13 (5.9%)	**<0.001**
EHRA class				
EHRA 1	126 (42.6%)	16 (21.1%)	110 (50.0%)	**<0.001**
EHRA 2a	150 (50.7%)	51 (67.1%)	99 (45.0%)	**<0.001**
EHRA 2b	16 (5.4%)	7 (9.2%)	9 (4.1%)	**<0.001**
EHRA ≥ 3	3 (1.0%)	2 (2.6%)	1 (0.5%)	**<0.001**
Left atrium size (mm)				
<40	19 (6.4%)	9 (11.8%)	10 (4.5%)	0.147
40–49	148 (50.0%)	38 (50.0%)	110 (50.0%)	0.147
50–59	108 (36.5%)	28 (36.8%)	80 (36.4%)	0.147
≥60	8 (2.7%)	1 (1.3%)	7 (3.2%)	0.147
Unknown	13 (4.4%)	-	13 (5.9%)	
Arterial hypertension	235 (79.4%)	60 (78.9%)	175 (79.5%)	1.00
Diabetes mellitus	44 (14.9%)	9 (11.8%)	35 (15.9%)	0.50
Chronic kidney disease	69 (23.3%)	10 (13.3%)	59 (26.8%)	**0.02**
OSA	12 (4.1%)	3 (3.9%)	9 (4.1%)	1.00
CHA_2_DS_2_-VA	2.7 ± 1.4	2.6 ± 1.3	2.7 ± 1.3	0.65
Heart failure				
No	141 (47.7%)	35 (46.1%)	106 (48.1%)	0.85
HFrEF	43 (14.5%)	9 (11.8%)	34 (15.5%)	0.85
HFpEF	74 (25%)	23 (30.3%)	51 (23.2%)	0.85
HFmrEF	24 (8.1%)	6 (7.9%)	18 (8.2%)	0.85
HFimpEF	14 (4.7%)	3 (3.9%)	11 (5.0%)	0.85
Smoking				
Yes	51 (17.2%)	14 (18.4%)	37 (16.8%)	0.79
No	198 (66.9%)	58 (76.3%)	140 (63.6%)	0.79
Unknown	47 (15.9%)	4 (5.3%)	43 (19.5%)	0.79
Successful eECV	278 (93.9%)	72 (94.7%)	206 (93.6%)	0.711

**Table 4:** Anticoagulation therapy at the time of left atrial appendage thrombus detection.

Anticoagulant	n (%)
Apixaban 5 mg twice daily	2 (22.2)
Apixaban 2.5 mg twice daily	1 (11.1)
Rivaroxaban 20 mg once daily	2 (22.2)
Rivaroxaban 15 mg once daily	2 (22.2)
Dabigatran 150 mg twice daily	1 (11.1)
Edoxaban 60 mg once daily	1 (11.1)

## Data Availability

Original data are available upon request.

## Abbreviations

The following abbreviations are used in this manuscript:

AF	atrial fibrillation
BMI	body mass index
CI	confidence interval
CrCl	creatinine clearance
DOAC	direct oral anticoagulant
ECV	electrical cardioversion
eECV	elective electrical cardioversion
EHRA	European Heart Rhythm Association
HF	heart failure
HFpEF	heart failure with preserved ejection fraction
HFmrEF	heart failure with mildly reduced ejection fraction
HFrEF	heart failure with reduced ejection fraction
INR	international normalized ratio
LA	left atrium
LAA	left atrial appendage
OAC	oral anticoagulation
OSA	obstructive sleep apnea
RR	relative risk
SD	standard deviation
TE	thromboembolic event
TEE	transesophageal echocardiography
